# The Microbiota–Endometriosis Axis: An Immune–Endocrine Integration Model and Emerging Therapeutic Targets

**DOI:** 10.3390/ijms27114883

**Published:** 2026-05-28

**Authors:** Georgiana Nemeti, Anca Elena Crăciun, Dan Claudiu Măgureanu, Florentina Claudia Militaru, Ioana Corina Bocsan, Cristian-Ioan Crăciun, Adriana Rusu, Carmen Stanca Melincovici, Anca Dana Buzoianu, Daniel Mureșan, Maria Adriana Neag

**Affiliations:** 1 Department of Obstetrics and Gynecology, “Iuliu Hațieganu” University of Medicine and Pharmacy, 400015 Cluj-Napoca, Romania; georgiana.nemeti@elearn.umfcluj.ro (G.N.); muresandaniel01@elearn.umfcluj.ro (D.M.); 2 2nd Department of Faculty of Nursing and Health Sciences, “Iuliu Hațieganu” University of Medicine and Pharmacy, 2-4 Clinicilor St., 400006 Cluj-Napoca, Romania; adriana.rusu@umfcluj.ro; 3 Department of Diabetes and Nutrition Diseases, Emergency Clinical County Hospital Cluj, 400006 Cluj-Napoca, Romania; 4 Department of Pharmacology, Toxicology and Clinical Pharmacology, “Iuliu Hațieganu” University of Medicine and Pharmacy, 400012 Cluj-Napoca, Romania; dan.clau.magureanu@elearn.umfcluj.ro (D.C.M.); claudia.militaru@umfcluj.ro (F.C.M.); bocsan.corina@umfcluj.ro (I.C.B.); cristian.craciun@umfcluj.ro (C.-I.C.); abuzoianu@umfcluj.ro (A.D.B.); maria.neag@umfcluj.ro (M.A.N.); 5 Discipline of Histology, Department of Morpho-Functional Sciences, “Iuliu Hațieganu” University of Medicine and Pharmacy, 400349 Cluj-Napoca, Romania; carmen.melincovici@umfcluj.ro; 6 Department of Healthcare-Associated Infections, Emergency Clinical County Hospital Cluj, 400006 Cluj-Napoca, Romania

**Keywords:** endometriosis, microbiota, estrogen metabolism, immune dysregulation, dysbiosis, inflammation, probiotics

## Abstract

Endometriosis is a chronic, estrogen-dependent inflammatory disorder characterized by the ectopic implantation and persistence of endometrial-like tissue outside the uterine cavity. Despite its high prevalence and significant impact on quality of life, the pathogenesis of endometriosis remains incompletely understood and involves a complex interplay between hormonal dysregulation, immune dysfunction, and chronic inflammation. In recent years, growing evidence has highlighted the role of the microbiota as a potential modulator of these interconnected pathways. This review proposes an integrative framework in which the microbiota acts as a central modulator of immune–endocrine interactions in endometriosis, while synthesizing current evidence on underlying biological mechanisms. We discuss how alterations in the gut, vaginal, and endometrial microbiota contribute to disease pathophysiology through multiple mechanisms, including disruption of intestinal barrier integrity, activation of pro-inflammatory signaling pathways, immune dysregulation, and modulation of estrogen metabolism via the estrobolome. Microbial β-glucuronidase activity and enterohepatic recirculation of estrogens are explored as key processes linking gut dysbiosis to the hyperestrogenic environment characteristic of endometriosis. Furthermore, we review current pharmacological treatments and highlight their limitations, emphasizing the need for novel therapeutic strategies targeting upstream disease mechanisms. Emerging approaches, including probiotics, postbiotics, short-chain fatty acids, and dietary interventions, are discussed as promising adjunctive therapies capable of modulating inflammation, immune responses, and metabolic pathways. Although current evidence remains heterogeneous and largely derived from preclinical and observational studies, the microbiota emerges not only as a potential therapeutic target but as a key integrative node linking endocrine, immune, and metabolic pathways in endometriosis. Future research should focus on well-designed clinical trials to validate microbiome-based interventions and to define their role in personalized management strategies for endometriosis.

## 1. Introduction

Endometriosis is a female-specific, estrogen-driven, often progressive, chronic inflammatory disorder in which endometrial-like cells and/or stroma are dispersed outside their normal location, the uterine cavity [1,2]. The pathogenesis is unclear, and the ectopic growth of the cells takes place frequently on the pelvic peritoneal surface, invading pelvic organs and structures such as the ovaries, bladder, rectum, retrovaginal septum, and, in rare cases, also affecting the pleura, pericardium, and diaphragm [3]. When the growth takes place within the uterine myometrium, it is called adenomyosis [4]. It is estimated that 1 out of 10 premenopausal women has endometriosis [5]. Despite this high prevalence and substantial disease burden, endometriosis remains underprioritized in awareness and research funding, as reflected in advocacy initiatives such as the European Endometriosis Alliance and in the limited European Union investment in endometriosis research to date [6,7].

Endometriosis is increasingly recognized as a systemic disease driven by complex interactions between hormonal, immune, and inflammatory pathways. Recent evidence highlights the microbiota as a potential regulator of these processes, influencing immune responses and estrogen metabolism. This perspective supports the conceptual framework of the present review, which positions the microbiota as an integrative factor in disease pathophysiology [6,7].

The clinical presentation of endometriosis is polymorphic. It can include fatigue, infertility, dysmenorrhea, dyspareunia, dysuria, and/or cyclical hematuria, dyschezia, painful rectal bleeding, other digestive symptoms (constipation, diarrhea, postprandial bloating), and pain of variable intensity (usually linked to the menstrual cycle). In some cases, the patient presents with cyclical chest pain, hemoptysis, cough, or catamenial pnemothorax [2]. Alongside physical symptoms, endometriosis is also a driver for psychological manifestations, such as anxiety and depression, interfering with social relationships [8]. Up to 20–25% of the patients have no symptoms, but 35–50% of reproductive-age women with pelvic pain and/or infertility have endometriosis [9]. Endometriosis can be diagnosed by imaging or by laparoscopy, and clinical evaluation should guide the choice between them. A normal pelvic examination does not exclude the disease. Transvaginal ultrasound is the first-line imaging modality, with MRI as an adjunct when ultrasound is inconclusive or for mapping deep disease; when positive, imaging is sufficient to diagnose ovarian endometrioma and deep infiltrating endometriosis without the need for surgical confirmation. However, imaging can be negative, particularly in superficial peritoneal disease, which it cannot reliably detect, so a negative scan does not rule out endometriosis. Laparoscopy is no longer required to establish the diagnosis and is reserved for women with negative or inconclusive imaging in whom symptoms persist, or when surgery is indicated for treatment; when lesions are excised, histological examination provides confirmation, although negative histology does not exclude the disease. The empirical treatment with hormonal therapy can be considered in suspected cases of endometriosis without laparoscopic/imaging confirmation of the diagnosis. We do not yet have validated biomarkers from blood, uterine, and/or menstrual fluids to diagnose endometriosis [2]. The mean time elapsed from the onset of the symptoms and the diagnosis is around 7 years, up to 12 years in younger women (under 19 years) and 3 years in women over 30 years. The diagnosis is established sooner in women with infertility as the main complaint (4 years) versus women with pain (7.4 years) [10]. Does early diagnosis of endometriosis versus late diagnosis lead to better quality of life? is a question from the 2022 ESHRE Guideline: Endometriosis, and the conclusion is that there are no data to support the benefits of early diagnosis, but should attempt to offer treatment to symptomatic women in order to relieve symptoms [2].

Endometriosis pathophysiology is similar to other chronic inflammatory diseases, such as autoimmune diseases or diabetes mellitus, being the result of dysregulated cross-talk between genetics/epigenetics, immunological, hormonal, and environmental factors [11]. The endometrioma, unlike other benign cysts, does not have a capsule. The reactive oxygen species and pro-inflammatory cytokines produced in the endometrioma can easily diffuse into the surrounding tissues and induce oxidative stress and fibrosis [12]. Furthermore, free iron is increased in the tissues in the proximity of endometrioma, with direct effect on lipid peroxidation, oxidative stress, and inflammation [13]. The microenvironment of the endometrioma shares similarities with the tumoral microenvironment: the presence of IL-1, IL-6, IL-8, and tumor necrosis factor-α (TNF-α), growth factors, hypoxia, prostaglandins, and ROS. It was also observed that a higher number of exhausted natural killer (NK) cells was found in the peritoneal fluid, but not in peripheral blood, in women with endometriosis [14]. The systemic inflammation modulation plays an important role in the development and progression of endometriosis. The plasma from patients with endometriosis has higher levels of proinflammatory cytokines compared with controls, and removal of endometriomas reduces the inflammation in the acute state, but this situation is transient. Interestingly, after 3 months from the removal of the ectopic lesion, it was observed that the inflammation starts to increase in the majority of the cases, reflecting the influence of the systemic factors [15]. The increased level of IL-10 observed in the serum of women with endometriosis may contribute to the development of endometriosis by suppressing immunity against endometrial implants [16]. The locally produced factors induce a generalized B-cell activation and increase the autoimmune response [17]. In the Nurses’ Health Study II, an association between endometriosis and systemic lupus erythematosus and rheumatoid arthritis was observed [18]. The role of microbiota in endometriosis as a disruptor of immune normal function is a relatively new concept [19].

Beyond these established mechanisms, emerging evidence suggests that endometriosis may not be solely a localized gynecological disorder, but rather a systemic condition shaped by complex interactions between endocrine, immune, and metabolic pathways. In this context, the microbiota has gained increasing attention as a potential integrative regulator capable of simultaneously modulating inflammation, immune responses, and estrogen metabolism. This perspective introduces a novel conceptual framework in which microbiota-driven processes may contribute to disease heterogeneity, persistence, and variable therapeutic response. The aim of this review is to integrate current evidence on the biological foundations of endometriosis with emerging insights into the microbiota–endometriosis axis, and to propose a unifying model in which microbiota-driven immune and endocrine interactions contribute to disease pathogenesis and progression. Additionally, we examine current and emerging therapeutic strategies, with a particular focus on microbiota-targeted interventions as potential modulators of disease mechanisms.

This narrative review was conducted using a structured literature search to summarize current evidence on the microbiota–endometriosis axis and its immune–endocrine interactions. A comprehensive search was performed in PubMed/MEDLINE, Scopus, and Web of Science for studies published up to March 2026, using keywords such as “endometriosis”, “microbiota”, “estrobolome”, “immune dysregulation”, “estrogen metabolism”, “dysbiosis”, “probiotics”, and “postbiotics”. Boolean operators were applied to refine the search. Additionally, reference lists of relevant articles were manually screened. Eligible studies included original articles, systematic reviews, and meta-analyses addressing the role of microbiota in endometriosis pathophysiology, as well as its immune, endocrine, and metabolic interactions. Both preclinical and clinical studies were considered. Non-English articles and studies not directly relevant to the microbiota–endometriosis axis were excluded. Data were synthesized qualitatively, focusing on key thematic areas: biological mechanisms, microbiota-related pathways, and therapeutic strategies. Given the heterogeneity of the available evidence, this review provides an integrative and critical perspective rather than a quantitative analysis.

## 2. Biological Foundations of Endometriosis

Endometriosis is a chronic, estrogen-dependent inflammatory disease defined by ectopic implantation and survival of endometrial-like tissue outside the uterine cavity. Although retrograde menstruation occurs in most menstruating women, only a subset develops the disease, indicating that additional endocrine, immune, and molecular abnormalities are required for lesion establishment and progression [5,20]. Rather than reflecting a single pathogenic trigger, endometriosis arises from a tightly interconnected network of local estrogen excess, progesterone resistance, immune dysfunction, chronic inflammation, angiogenesis, and fibrosis, which together sustain lesion persistence [21]. The principal mechanisms and key molecular players are summarized in Table 1; below, we briefly highlight the features most relevant for the microbiota-centered model developed in subsequent sections.

Local estrogen excess is a defining feature: endometriotic stromal cells aberrantly express aromatase (CYP19A1) and steroidogenic acute regulatory protein (StAR), enabling intracrine conversion of circulating androgens to estradiol, while expression of 17β-hydroxysteroid dehydrogenase type 2 (17β-HSD2), which inactivates estradiol to estrone, is markedly reduced [5,22,23,24,25,26,27,28]. This intracrine activity is reinforced by estrogen receptor (ER) imbalance, with epigenetically driven ERβ overexpression and relative ERα suppression; the resulting aberrant ERβ/ERα ratio promotes lesion survival, induces COX-2 and PGE2, and reciprocally amplifies aromatase expression, establishing a self-sustaining feed-forward loop [29,30,31,32,33]. Crucially for the present review, this loop operates within an enterohepatic estrogen pool whose magnitude is modulated by gut microbial β-glucuronidase activity (the estrobolome), providing a mechanistic link to dysbiosis (Section 3 and Section 4).

Progesterone resistance compounds this estrogen-dominant state. Reduced progesterone receptor B (PR-B) expression, with relative PR-A predominance, prevents normal progesterone-mediated induction of 17β-HSD2 and HOXA10 and fails to suppress NF-κB, sustaining inflammatory signaling [5,21,34,35,36]. Immune dysfunction is the second pillar relevant to the microbiota interface: NK cell cytotoxicity is impaired, peritoneal macrophages shift toward a pro-inflammatory, tissue-remodeling phenotype that secretes TNF-α, IL-1β, IL-6, and VEGF, and PD-1/PD-L1 checkpoint activation reinforces immune tolerance toward ectopic cells [5,37,38,39,40,41,42,43,44,45]. These immune alterations are precisely the targets through which gut and reproductive-tract dysbiosis are hypothesized to act, via Toll-like receptor (TLR) signaling, lipopolysaccharide (LPS) exposure, and short-chain fatty acid (SCFA) availability (Section 4).

The downstream consequences, chronic inflammation, angiogenesis, and fibrosis, are also relevant. Sustained NF-κB and MAPK activation, elevated IL-1β, IL-6, TNF-α, CCL2, and CCL5 in peritoneal fluid, and an estradiol-driven COX-2/PGE2 amplification loop maintain a permissive inflammatory milieu and sensitize peripheral and central pain pathways [46,47,48,49,50]. Hypoxia-induced HIF-1α activation, macrophage-derived VEGF, and IL-17A converge on pathological neovascularization [51,52,53,54,55,56,57,58]. TGF-β, activin A, CTGF, and S1P drive epithelial-to-mesenchymal, fibroblast-to-myofibroblast, and macrophage-to-myofibroblast transitions, with progressive extracellular matrix deposition particularly evident in deep infiltrating disease [59,60,61,62,63,64,65,66]. Lesion persistence and recurrence are additionally supported by endometrial mesenchymal stem/stromal cells (eMSCs), which display enhanced migratory and adhesive behaviors and contribute directly to fibrotic remodeling [20,67,68,69,70].

Several of these pathways, particularly TLR-mediated inflammation, NK-cell and macrophage function, estrogen metabolism, and barrier integrity, are now recognized to be influenced by the microbiota. This convergence provides the mechanistic basis for an integrative microbiota–immune–endocrine model of endometriosis, formalized in the following sections.

## 3. Microbiota in Reproductive and Systemic Homeostasis

The reproductive system and the gut are hosting a diverse collection of microorganisms (e.g., bacteria, viruses, fungi) which compose the microbiota. All the genetic material forms a microbiome, and the genes involved in estrogen metabolism (from gut bacteria) form the estrobolome [19].

The vaginal microbiota is a dynamic system that is in continuous change, according to the menstrual cycle, other internal factors (pregnancy, age, race, immune system function, chronic stress), and external factors (humidity, temperature, douching, infections, antibiotics). The vaginal microbiota of a healthy woman of reproductive age is composed of aerobic and anaerobic species, which are the source of various products, including antimicrobial compounds, and induce the low-level immune system activation, forming the first barrier against pathogen agents [71]. The “healthy” landscape is dominated by *Lactobacillus* spp. Altered microbiota produces bacterial vaginosis, characterized by the reduction of *Lactobacillus* and an increase in anaerobic bacteria such as *Gardnerella* or *Mobiluncus* spp. The prevalence of bacterial vaginosis (BV) is estimated to be 23–29% among women of reproductive age [72] and is associated with pelvic inflammatory disease, reduced resistance to sexually transmitted diseases, infertility, and adverse pregnancy outcomes [73]. The diagnosis of BV is performed using Amsel criteria: (1) homogenous, thin, white-yellow vaginal discharge; (2) clue cells on saline wet mount microscopy; (3) a vaginal fluid pH > 4.5; (4) positive result of whiff-amine test (release of a fishy odor after adding 10% solution of potassium hydroxide to wet mount). Any 3 positive criteria present confirm the diagnosis. The Nugent scoring system adds more specific details about the presence of three morphotypes: *Lactobacillus*, *Gardnerella*, and curved gram rods; a score of more than 7 (out of 10) is positive for BV [74].

The characterization of vaginal bacteria composition was performed in a group of 396 healthy women of reproductive age representing different ethnic groups using pyrosequencing of barcoded 16S rRNA genes. There were five clusters identified: cluster 1 dominated by *Lactobacillus iners*, cluster 2—*Lactobacillus crispatus*, cluster 3—*Lactobacillus gasseri*, cluster 4—low proportions of *Lactobacillus* spp. and high proportions of anaerobic organisms, and cluster 5 dominated by *Lactobacillus jensenii*. There was a different proportion of clusters according to ethnic groups; more than 80% of Asian and white women had a cluster dominated by *Lactobacillus* spp. and a low vaginal pH, while around 60% of black and Hispanic women had a cluster dominated by anaerobic bacteria and higher vaginal pH [75]. These findings challenge the perception of a healthy vaginal microbiome being dominated by *Lactobacillus* and pH below 4.5 as a one-size-fits-all standard, promoting the idea of refining the estimation of bacterial vaginosis risk according to ethnicity as a first step for personalized medicine and underline the importance of behavioral, cultural and biodiverse factors linked to the host lifestyle that might have a role in shaping the vaginal microbiome composition [71].

The endometrial microbiome is an emerging topic in the field of the reproductive health. The role of bacteria colonizing the endometrial surface is poorly understood and the methodology of sampling is important in order to minimize contamination. In a recent published study, the sampling of endometrial fluid using double-lumen catheters (used for embrio-transfer) from 53 women scheduled for in vitro fertilization procedures showed interesting results [76]. The samples were analysed by V3-V4-V6 regions of 16 rRNA gene sequencing with Next-Generation Sequencing technique and indicate that only 4 women (8%) had endometrial microbiome dominated by *Lactobacillus* spp. and also only 8% of the cases had similarities between vaginal and endometrial microbiome composition. Although there was no microorganism identified to be associated with failure to obtain pregnancy, the successful cases had a significant higher diversity score of endometrial microbiome [76].

The gut metabolome plays a role in the modulation of estrogen metabolism through the estrobolome, which is formed by the bacterial genes that encodes enzymes involved in the estrogen metabolism, such as β-glucosidase and β-glucuronidase [77]. The estrogen is inactivated in the liver by conjugation, but β-glucuronidase secreted by gut bacteria can deconjugate the inactive form and reactivate the estrogen. The role of gut microbial β-glucuronidase (gmGUS) as an important regulator in estrogen metabolism has been established [78]. The Human Microbiome Project GI database identified 279 unique gmGUS proteins clusters into six structural categories. According to the abundance of gmGUS, the following phyla were observed: *Bacteroidetes* (52%—contains proteins from 5 out of 6 clusters, including some rare forms), *Firmicutes* (43%), *Verrucomicrobia* (1.5%—4 proteins from the same cluster), and *Proteobacteria* (0.5%—a single gmGUS) [79]. The role of gut dysbiosis in the pathology might be explained by the decrease in deconjugation of estrogen, resulting low circulating levels. This mechanism is involved also in the development of obesity, metabolic syndrome, or cardiovascular disease [80].

The endocrine disruptors can play a negative role by direct effect on gut bacteria, or indirect effect by interfering with hormonal synthesis or receptor activation. A number of 450 compounds, including endocrine disruptors, have estrogenic activity that impacts the reproductive function both in males and females [81]. One example is bisphenol A (BPA), with estrogenic effects on parents and offspring in a rodent model, with changes of the microbiota towards a specific one to female sex [82]. On the other hand, the serum concentration of BPA in women with polycystic ovary syndrome is higher than in healthy women, but with no established causality [83].

**Figure 1:**
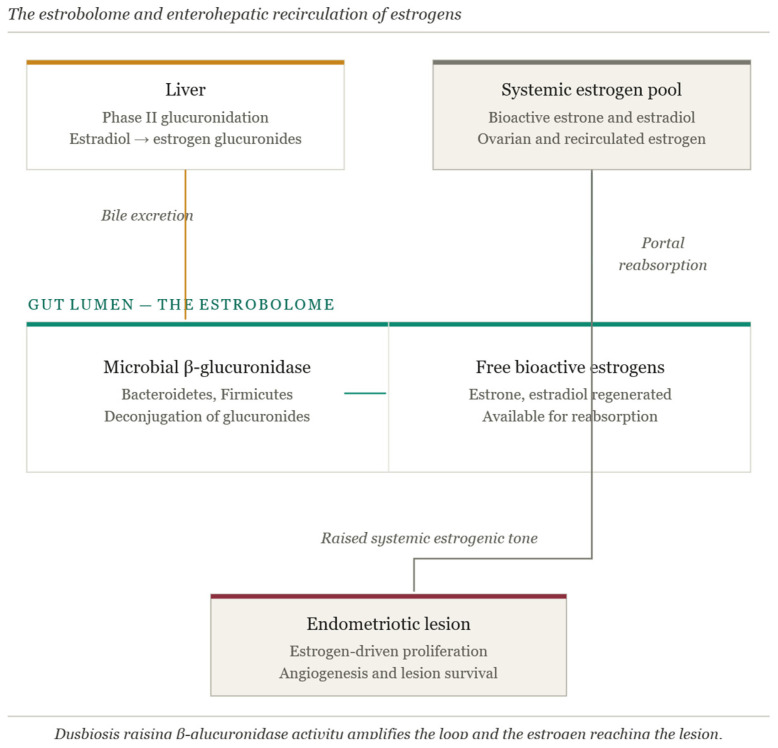
Estrobolome—gut microbiota relationship.

The relationship between estrogen metabolism and the gut microbiota is bidirectional (Figure 1) [84]. The microbiota-to-host arm acts predominantly through β-glucuronidase-mediated deconjugation of conjugated estrogens excreted in bile, regenerating bioactive estrone and estradiol that re-enter the enterohepatic circulation and thereby modulate systemic estrogenic tone. The reciprocal host-to-microbiota arm is mechanistically distinct and operates through at least four converging routes. First, estrogens modulate mucosal immunity: estrogen receptors are expressed on enterocytes and on lamina propria immune cells, and estrogen signaling enhances secretory IgA transcytosis across the intestinal epithelium, thereby shaping which microbial taxa are tolerated, opsonized, or excluded. Second, estrogens influence the chemical and physical properties of the mucus layer through effects on goblet cell function and mucin glycosylation, modifying habitat availability for mucin-degrading and mucin-adherent taxa. Third, estrogens regulate intestinal transit and motility via effects on enteric neurons and smooth muscle, which alter luminal residence time, substrate availability, and consequently community composition. Fourth, estrogens contribute to the maintenance of epithelial barrier integrity and to local pH and bile-acid pool composition, all of which exert ecological selection pressures on the resident community. Through this combination of immunological, mucosal, motility-related, and metabolic effects, host estrogenic status shapes microbial diversity and the relative abundance of taxa encoding β-glucuronidase activity, closing a bidirectional loop in which microbiota and estrogens regulate each other [84].

Collectively, these observations suggest that the microbiota plays a central role in maintaining reproductive and systemic homeostasis through its effects on barrier integrity, immune regulation, and estrogen metabolism. These mechanisms are particularly relevant in the context of endometriosis, where immune dysfunction, chronic inflammation, and estrogen dependence represent core features of disease pathophysiology.

## 4. Microbiota and Endometriosis: Current Evidence

The gut microbiota is a complex ecosystem that is essential for preserving intestinal barrier integrity through various mechanisms, such as the production of antimicrobial peptides and short-chain fatty acids (SCFAs), as well as the regulation of tight junction proteins [85]. Commensal microbes of the gut microbiota maintain intestinal homeostasis by inhibiting pathogen colonization, modulating immune responses, and strengthening the gut barrier through Toll-like receptor activation, mucus and antimicrobial protein production, and short-chain fatty acid synthesis, whereas dysbiosis disrupts these processes and exerts detrimental effects on the host [86,87]. A reduction in protective anaerobic bacteria, particularly *Lactobacillus* and *Bifidobacterium* species, together with the overgrowth of pathobionts such as *Bacteroides*, *Prevotella*, and *Escherichia*, disrupts epithelial permeability and undermines intestinal homeostasis [88,89]. Dysbiosis, the alteration of the composition and diversity of the human microbiome (gut, vaginal, and endometrial), may be an important factor contributing to the development and severity of endometriosis. In the cervical and endometrial compartments, *Gardnerella*, *Streptococcus*, *Escherichia*, *Shigella*, and *Ureaplasma* have been reported at increased abundance in women with stage 3/4 endometriosis relative to controls, whereas *Shigella*/*Escherichia* dominance has been observed in the gut (stool) microbiome of the same population [90]. A reciprocal reduction in beneficial bacteria, including *Lactobacillus*, *Bifidobacterium*, and *Ruminococcaceae*, has been described across gut and reproductive-tract samples [90,91].

The transition from eubiosis to dysbiosis triggers multiple pathways that increase intestinal permeability. Dysbiotic microbiota induce epithelial barrier disruption through several mechanisms: disruption of tight junction protein expression and localization, increased epithelial cell apoptosis, enhanced paracellular transport, and altered transcellular permeability [92].

### 4.1. Gut Barrier Dysfunction, Bacterial Translocation, and Peritoneal Inflammation

In dysbiosis, increased TLR signaling, notably through bacterial lipopolysaccharide (LPS) engagement of TLR4, drives production of TNF-α, IL-6, and IL-1β, which in turn disrupt tight junction proteins, upregulate zonulin (prehaptoglobin-2), a physiological regulator of paracellular permeability acting through PAR2/EGFR transactivation and PKC-mediated reorganization of claudins, occludin and zonula occludens proteins [93,94,95,96], and amplify regulated forms of epithelial cell death, including apoptosis, necroptosis, pyroptosis, and ferroptosis [96,97,98,99,100,101,102,103,104,105,106]. The net result is a “leaky” gut barrier permitting translocation of microbial antigens and endotoxins into the submucosa and, ultimately, into the peritoneal cavity [92,107,108,109,110,111]. In endometriosis, LPS is detected at elevated concentrations in peritoneal fluid and is the principal mediator stimulating peritoneal macrophages to produce hepatocyte growth factor (HGF), VEGF, IL-6, and TNF-α, cytokines and growth factors that promote lesion implantation and progression in the pelvic microenvironment [112]. Although the upstream events, zonulin signaling and regulated epithelial cell death, are not unique to endometriosis, the consequence, LPS-driven peritoneal inflammation, links gut barrier dysfunction directly to the mechanisms that support ectopic lesion survival.

### 4.2. Microbiota and Estrogen Metabolism

The gut microbiome is a key regulator of estrogen metabolism via a distinct set of bacterial genes collectively termed the estrobolome. These genes encode enzymes such as β-glucuronidases and β-glucosidases, which control circulating estrogen levels and contribute to the development of estrogen-dependent conditions, including endometriosis [77,78,113,114].

Estrogens undergo hepatic phase II metabolism through glucuronidation, forming inactive estrogen glucuronides that are excreted into the intestinal lumen via bile. Within the gastrointestinal tract, microbial β-glucuronidase enzymes mediate the deconjugation of these glucuronides, regenerating bioactive estrogens such as estrone and estradiol. This microbial-mediated deconjugation facilitates reabsorption of free estrogens via the enterohepatic circulation, thereby augmenting systemic estrogen bioavailability and potentially influencing estrogen-dependent pathophysiology [115,116,117]. Experimental studies have identified that distinct classes of gut microbial β-glucuronidase enzymes, specifically Loop 1, mini-Loop 1, and FMN-binding variants, are capable of reactivating estrogen glucuronides. The efficiency of this enzymatic reactivation is contingent upon the composition and functional potential of the gut microbiome, highlighting a bidirectional interplay between microbial diversity and systemic estrogen homeostasis [115].

Disruption of the gut microbial β-glucuronidase-estrogen axis due to dysbiosis can lead to dysregulated estrogen metabolism, potentially contributing to the onset and progression of estrogen-dependent disorders [80,118]. In women with endometriosis, alterations in the gut microbiome and estrobolome have been reported, although findings across studies remain heterogeneous. A case-control study demonstrated that fecal samples from endometriosis patients exhibited enrichment of specific bacterial taxa and elevated levels of estrogen and estrogen metabolites, despite no significant differences in overall β-glucuronidase activity or microbial diversity. Additionally, increased inflammatory markers, including β-glucuronidase and secretory IgA, have been observed in these patients, suggesting a mechanistic link between gut microbial composition, estrogen metabolism, and systemic inflammation [119,120].

In addition to modulating endogenous estrogen levels, the gut microbiota plays a critical role in metabolizing dietary phytoestrogens, thereby influencing systemic estrogenic activity. Isoflavones, lignans, and coumestans, major classes of phytoestrogens found in soy, flaxseed, and other plant-based foods, are metabolized by specific gut bacteria into bioactive compounds with varying affinities for estrogen receptors [121,122]. For instance, the isoflavone daidzein can be converted by equol-producing bacteria into equol, a metabolite exhibiting higher estrogenic potency and preferential binding to estrogen receptor β compared to its precursor [121,123]. Similarly, plant lignans are metabolized into enterolignans (enterodiol and enterolactone), which have mild estrogenic or anti-estrogenic properties. Importantly, the ability to produce equol or enterolignans varies markedly among individuals and populations, largely depending on the presence and activity of specific bacterial species in the gut microbiome. This interindividual variability may influence the systemic estrogenic milieu and modulate the risk or progression of estrogen-dependent disorders, including endometriosis, breast cancer, and menopausal symptoms [122,124,125]. Furthermore, dietary patterns, antibiotic use, and overall gut microbial diversity can affect the efficiency of phytoestrogen metabolism, highlighting the intricate interplay between diet, microbiota composition, and host hormonal status [126].

The relationship between gut dysbiosis and endometriosis appears to be bidirectional. Animal models indicate that endometriotic lesions can induce changes in gut microbiota, while microbiota alterations can, in turn, promote disease initiation and progression [126,127,128]. Recent studies further highlight specific pathogenic mechanisms, including the translocation of gut bacteria, particularly Pseudomonas species, into the peritoneal cavity, where they may trigger local inflammatory cascades that facilitate the development and growth of endometriotic lesions [129].

### 4.3. An Integrative Immune–Endocrine Model of the Microbiota–Endometriosis Axis

**Figure 2:**
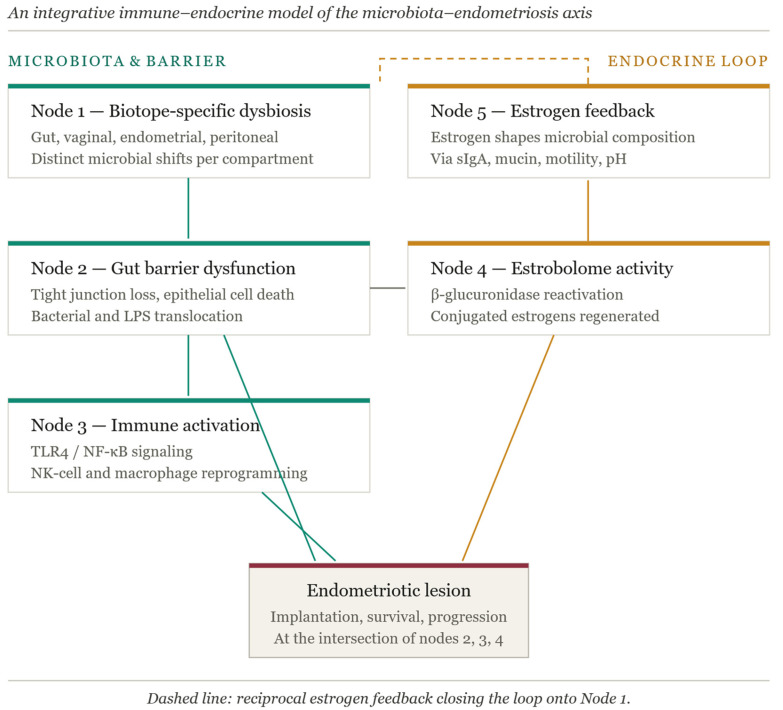
Proposed five-node immune–endocrine integration model of the microbiota–endometriosis axis. The model links microbial, barrier, immune, and endocrine mechanisms into a self-reinforcing loop, with the endometriotic lesion situated at the intersection of Nodes 2, 3, and 4. Solid arrows indicate the dominant direction of influence; the dashed arrow denotes the reciprocal estrogen feedback that completes the cycle. Abbreviations: LPS, lipopolysaccharide; NF-κB, nuclear factor kappa B; sIgA, secretory immunoglobulin A; TLR4, Toll-like receptor 4.

Multiple mechanisms link microbiota-driven alterations to endometriosis pathogenesis [19,130]. We integrate them here, proposing a five-node model in which microbial dysbiosis acts as a central modulator of immune and endocrine pathways (Figure 2).

Node 1—Immune dysregulation. Gut dysbiosis disrupts immune homeostasis by altering host–microbe interactions, leading to excessive activation of innate and adaptive immune responses. This process is characterized by increased production of proinflammatory cytokines, such as IL-1β, IL-6, IL-17, and TNF-α, together with impaired immunosurveillance resulting from dysfunctional macrophage, natural killer (NK), and T cell activity [131,132]. Moreover, dysbiosis-induced shifts in immune cell populations, including an increased Th17/Treg ratio and altered macrophage polarization, create a chronic inflammatory and immune-permissive microenvironment [133]. These immune alterations reduce the clearance of ectopic endometrial cells and promote their adhesion, survival, and angiogenesis within the peritoneal cavity, thereby facilitating the establishment and progression of endometriotic lesions [134,135].

Node 2—Bacterial translocation and LPS–TLR4 signaling. Chronic inflammation and bacterial translocation play a crucial role in the establishment, maintenance, and progression of ectopic endometrial lesions. In addition to dysregulated immune cell activity and elevated pro-inflammatory cytokines, increasing evidence implicates bacterial endotoxins, particularly lipopolysaccharide (LPS), in sustaining the inflammatory milieu characteristic of the disease. LPS is detected at elevated levels in the peritoneal fluid of women with endometriosis and activates innate immune responses through Toll-like receptor 4 (TLR4) signaling. This activation promotes the production of inflammatory mediators such as TNF-α, IL-6, IL-8, prostaglandins, and reactive oxygen species, thereby enhancing macrophage activation, angiogenesis, and lesion survival. LPS-TLR4 signaling also contributes to progesterone resistance and altered endometrial receptivity, linking microbial-driven inflammation to both pain and infertility in endometriosis. Collectively, these findings support the concept that endometriosis represents, at least in part, a chronic inflammatory condition influenced by immune-microbial interactions, highlighting LPS and its downstream pathways as potential therapeutic targets. It is known that bacterial translocation represents a mechanistic bridge between gut dysbiosis, immune activation, and the progression of endometriotic lesions [129,136,137,138].

Node 3—Estrobolome-mediated estrogen reactivation. Altered estrogen signaling is also an important mechanism. Gut dysbiosis, characterized by shifts in microbial composition and estrobolome function, is associated with increased β-glucuronidase activity, leading to enhanced reactivation of conjugated estrogens and elevated circulating estrogen levels. This hyper-estrogenic environment accentuates the estrogen-dependent nature of endometriosis by promoting proliferation and angiogenesis of ectopic endometrial lesions and may also interact with immune and inflammatory pathways that support lesion survival and growth. Emerging clinical and mechanistic studies suggest that dysbiosis-induced estrobolome alterations could therefore contribute to hormonal imbalance and disease progression in endometriosis, offering a potential target for microbiome-based therapeutic strategies [139,140,141].

Node 4—Reciprocal estrogen-driven shaping of microbial composition. As detailed in Section 3, host estrogenic status modulates microbiota composition through secretory IgA transcytosis, mucin glycosylation, intestinal motility, and luminal pH and bile-acid composition. In the hyperestrogenic environment of endometriosis, these reciprocal effects can further entrench dysbiosis, creating a self-reinforcing pathological loop.

Node 5—Biotope-specific dysbiosis. Microbial alterations are not uniform across body sites: reported endometriosis-associated changes differ between the gut (stool), the cervical and endometrial compartments, and the peritoneal fluid, and these compartmental differences are central to the correct interpretation of any single bacterial association.

Together, these five interacting nodes constitute the immune–endocrine integration model proposed in this review. The model generates testable predictions: that gut barrier permeability markers will track peritoneal LPS concentrations; that estrobolome composition will predict circulating estrogen levels independently of ovarian status; that biotope-specific dysbiosis signatures will differ between superficial, ovarian, and deep infiltrating endometriosis; and that an intervention targeting any single node should produce measurable shifts in the others. Validation of these predictions in adequately powered human studies is a priority for the field.

## 5. Current and Emerging Therapeutic Strategies in Endometriosis: From Hormonal Suppression to Microbiota Modulation

**Figure 3:**
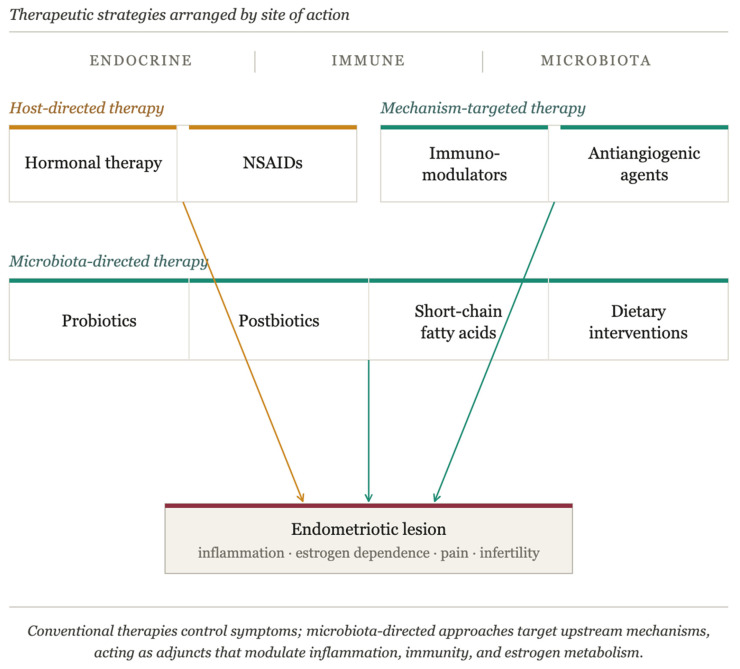
Therapeutic strategies landscape.

Current treatment strategies for endometriosis are primarily focused on symptom control rather than disease modification, largely relying on hormonal suppression and analgesic therapies. A structured summary of current and emerging therapeutic strategies, including their mechanisms of action, clinical effects, limitations, and key references, is presented in Figure 3, complemented by Table 2.

While these approaches can effectively reduce pain and lesion activity, they do not address the underlying mechanisms driving disease persistence, including immune dysfunction, chronic inflammation, and altered estrogen metabolism. This limitation has prompted increasing interest in therapeutic strategies targeting upstream regulatory pathways, including those modulated by the microbiota.

### 5.1. Nonsteroidal Anti-Inflammatory Drugs

Nonsteroidal anti-inflammatory drugs (NSAIDs) are widely recommended as first-line analgesic therapy for endometriosis-associated pain, often used alone or in combination with hormonal treatments. These medications exert their therapeutic effect by inhibiting COX enzymes, thereby reducing prostaglandin synthesis and inflammation, which are key mediators of pain in endometriosis [49,142].

NSAIDs may exert heterogeneous and target-dependent effects on disease biology. They were found to interact with multiple endometriosis-related molecular pathways, including angiogenesis and inflammatory signaling. Notably, EPHB4 emerged as a central risk-associated target bound by most NSAIDs, indicating a potential pro-angiogenic and disease-promoting effect. In contrast, indomethacin demonstrated a distinct dual-regulatory profile by interacting with both the risk-associated EPHB4 and the protective PTGER4 target, suggesting a more balanced molecular impact. These findings support a “double-edged sword” model of NSAID action in endometriosis and highlight the importance of target-oriented drug selection to optimize therapeutic efficacy while minimizing unintended disease-modifying effects [143].

Despite their widespread use, the evidence supporting the efficacy of NSAIDs for endometriosis pain is limited. A 2017 Cochrane systematic review identified only one low-quality trial involving 24 participants, which found no conclusive evidence that NSAIDs (naproxen) provided superior pain relief compared to placebo [142]. Nevertheless, NSAIDs remain a pragmatic first-line option due to their safety profile, accessibility, and tolerability, especially compared to hormonal agents, but escalation to hormonal therapy is appropriate if pain control is inadequate [144,145].

### 5.2. Hormonal Suppression

Hormonal suppression is the cornerstone of medical treatment for endometriosis-associated pain, targeting the sex steroid-dependent pathophysiology of this chronic inflammatory disease. All hormonal therapies work by suppressing ovarian activity and creating a hypoestrogenic environment, which induces regression of endometriosis lesions [49].

#### 5.2.1. Combined Estrogen-Progestin Contraceptives as First-Line Therapy

First-line treatment includes combined estrogen-progestin contraceptives (CEPCs) and progestin-only medications such as norethindrone acetate, recommended due to their low cost and favorable adverse effect profile. Estradiol promotes proinflammatory signaling and inhibits apoptosis in endometrial cells, particularly when they are ectopically implanted. In contrast, progestins suppress inflammatory pathways and responses while promoting apoptotic mechanisms in endometriotic cells [28,146]. Clinical studies have demonstrated that CEPCs effectively reduce dysmenorrhea, chronic pelvic pain, and dyspareunia in women with endometriosis, with a favorable safety profile and good long-term tolerability. Continuous or extended-cycle regimens are often preferred over cyclic regimens to minimize withdrawal bleeding and provide more consistent symptom relief. Despite their widespread use, CEPCs do not eliminate existing lesions, and symptom recurrence may occur after discontinuation, highlighting the need for individualized, long-term management strategies [147,148].

#### 5.2.2. Gonadotropin-Releasing Hormone Agonists and Antagonists as Second-Line Therapy

Gonadotropin-releasing hormone (GnRH) agonists and antagonists represent second-line hormonal therapies for endometriosis-associated pain, reserved for patients who fail to achieve adequate symptom control with first-line treatments such as combined hormonal contraceptives or progestins. They function through distinct but related mechanisms to achieve profound suppression of systemic estrogen levels [146]. The used GnRH agonists are leuprolide, goserelin, nafarelin, buserelin, and GnRH antagonists are elagolix, relugolix, linzagolix [149,150]. Nowadays, leuprolide acetate and nafarelin acetate are modified forms of native GnRH with prolonged half-lives that bind to pituitary GnRH receptors. Following initial administration, these agents produce a transient “flare effect” characterized by stimulation of gonadotropin release, but after approximately 10 days of continuous exposure, they induce downregulation and desensitization of pituitary GnRH receptors. This downregulation inhibits gonadotropin release and ovarian hormone secretion, ultimately creating a hypoestrogenic state [21,151]. GnRH antagonists function as competitive antagonists of pituitary GnRH receptors, blocking gonadotropin secretion within hours of administration. This rapid onset of action eliminates the initial flare effect observed with agonists and allows for dose-dependent modulation of estrogen suppression. The oral administration route of non-peptide GnRH antagonists offers significant advantages over injectable GnRH agonists, including improved convenience, rapid onset of action, and quick return of menses upon discontinuation (median time to resumption of menses is 31 days for relugolix combination therapy) [49,150,151,152,153].

So, like any therapeutic intervention, GnRH agonists and antagonists have significant advantages and disadvantages that must be carefully weighed when selecting second-line therapy for endometriosis. The disadvantages include a high incidence of hypoestrogenic adverse effects such as hot flushes, headache, mood changes, vaginal dryness, and, most importantly, bone mineral density loss, which may be irreversible with prolonged use without add-back therapy. These adverse effects limit the duration of therapy (typically ≤6 months for agonists without add-back, up to 12–24 months with add-back or for low-dose antagonists). Cost is substantially higher than that of first-line therapies, and long-term use is generally not feasible. Additionally, symptom recurrence is common after discontinuation, and these agents do not eliminate existing lesions [154,155,156].

#### 5.2.3. Aromatase Inhibitors as Alternative Therapy

Aromatase inhibitors (AIs) represent an alternative hormonal therapy for endometriosis-associated pain, particularly in patients who fail to respond to first- and second-line treatments or in postmenopausal women with symptomatic endometriosis. The rationale for using AIs in endometriosis is based on the observation that endometriotic lesions aberrantly express aromatase P450, the rate-limiting enzyme for estrogen biosynthesis, which is normally absent in healthy endometrium. This aberrant aromatase expression enables endometriotic lesions to produce estrogen locally and independently of ovarian function, creating a positive feedback loop that promotes lesion growth and inflammation [157,158,159]. Specifically, estradiol stimulates cyclooxygenase-2 (COX-2) expression, which increases prostaglandin E2 (PGE2) production; PGE2, in turn, is a potent inducer of aromatase activity in endometriotic tissue [160]. This self-perpetuating cycle of local estrogen production and inflammatory signaling drives the growth and persistence of ectopic endometrial tissue. By inhibiting aromatase, these agents decrease local estradiol production in endometriotic lesions, thereby interrupting this positive feedback loop and minimizing lesion growth and inflammatory signaling [159,161]. Despite their theoretical advantages and demonstrated efficacy in selected cases, the clinical use of AIs for endometriosis remains limited by several significant disadvantages. Common adverse effects include headaches, joint stiffness, leg cramps, hot flushes, and vasomotor symptoms similar to those experienced during menopause, which can significantly impact quality of life and treatment adherence. Long-term use is restricted by concerns about bone mineral density loss, particularly in premenopausal women, necessitating careful monitoring of bone health [151,162,163]. Table 3 summarizes the main drug classes used in the treatment of endometriosis.

#### 5.2.4. Metabolic Impact of Hormonal Therapies for Endometriosis: Effects on Glucose Metabolism, Weight, and Insulin Sensitivity

CEPCs can impact insulin sensitivity and glucose metabolism through both estrogenic and progestogenic components. CEPCs may modestly decrease insulin sensitivity and impair glucose tolerance, with the magnitude and clinical relevance of these effects depending largely on the type of progestin used in the formulation [171,172]. Estrogen, particularly ethinyl estradiol, increases hepatic production of binding proteins and can induce mild insulin resistance, while progestins modulate these effects [173,174]. Formulations containing androgenic progestins (e.g., levonorgestrel, norethindrone) are associated with greater reductions in insulin sensitivity and more pronounced adverse effects on glucose metabolism compared to those with antiandrogenic or neutral progestins (e.g., desogestrel, drospirenone, cyproterone acetate) [175,176]. Levonorgestrel-containing CEPCs, for example, have been shown to increase insulin resistance and second-phase pancreatic insulin secretion, while desogestrel-containing CEPCs may increase insulin half-life but also impair insulin sensitivity [177,178]. During long-term management, clinical considerations include monitoring for metabolic disturbances, especially in women with pre-existing risk factors for insulin resistance, obesity, or a family history of diabetes. The risk of clinically significant glucose metabolism disorders is low in healthy, reproductive-age women, but may be higher in perimenopausal women or those with metabolic syndrome. Selection of CEPCs with lower estrogen doses and non-androgenic progestins is preferred to minimize metabolic impact. Regular assessment of glucose and insulin parameters may be warranted in high-risk populations, and alternative therapies should be considered if metabolic derangements develop [172,179,180].

Regarding body weight, evidence suggests that CEPCs do not cause substantial weight gain, with systematic reviews finding no consistent association between combination contraceptives and weight change [172,181]. Progestin-only formulations exhibit variable metabolic effects that are highly dependent on both the specific progestin molecule and the route of administration. Structurally, progestins derived from testosterone (such as norethindrone, levonorgestrel) tend to have more androgenic activity, which can negatively impact lipid profiles and insulin sensitivity, whereas those with antiandrogenic or neutral properties (such as drospirenone, dienogest, nomegestrol acetate) are generally metabolically neutral or may even improve certain metabolic parameters [176,182,183]. The route of administration further influences these effects. Oral progestin-only pills (e.g., norethindrone, drospirenone) typically have minimal impact on glucose and lipid metabolism in healthy women, but individual agents differ: drospirenone is associated with anti-mineralocorticoid effects and may lower blood pressure, while norethindrone may have mild androgenic effects [184,185]. Injectable progestins, particularly depot medroxyprogesterone acetate (DMPA), are associated with increased insulin resistance and adverse changes in glucose metabolism, especially with long-term use [186,187]. In contrast, intrauterine systems (e.g., levonorgestrel IUS) generally have a more favorable metabolic profile, with minimal systemic effects [188].

Gonadotropin-releasing hormone (GnRH) agonists and antagonists induce metabolic changes primarily due to profound suppression of sex steroid hormones, resulting in a hypoestrogenic state. This leads to increased fasting insulin levels, reduced insulin sensitivity, and adverse lipid profiles, specifically elevated total cholesterol, LDL cholesterol, and triglycerides. These effects have been consistently observed in both men and women undergoing GnRH therapy for conditions such as prostate cancer, endometriosis, and uterine leiomyomas [189,190,191,192]. The mechanism underlying these changes is not primarily related to alterations in body composition, but rather to the direct impact of sex steroid deprivation on metabolic regulation. Short-term ovarian suppression with GnRH agonists in healthy women does not significantly alter body weight or fat distribution, yet still impairs insulin sensitivity and increases circulating lipids, supporting the concept that sex steroid deficiency itself drives these metabolic disturbances [193,194,195]. These metabolic effects are clinically relevant, as they contribute to increased risk of type 2 diabetes, atherosclerosis, and cardiovascular disease in patients receiving long-term GnRH therapy. The American Heart Association highlights the need for proactive cardiovascular risk management in adults treated with GnRH agonists or antagonists, including regular monitoring of glucose and lipid parameters and consideration of add-back therapy to mitigate hypoestrogenic side effects [191,196,197].

AIs, when used as third-line therapy for refractory endometriosis, are associated with increased insulin resistance and adiposity, as demonstrated in postmenopausal women with breast cancer. These metabolic changes are attributed to profound estrogen depletion, which disrupts glucose homeostasis and promotes adiposity. However, the clinical significance of these findings in endometriosis remains uncertain, as most metabolic data are derived from breast cancer populations rather than women with endometriosis [198]. Available evidence suggests that short-term use in endometriosis is generally well tolerated, but long-term safety data regarding metabolic effects are lacking [161,163]. Given these considerations, it is prudent to monitor glucose metabolism, weight, and cardiovascular risk factors in women receiving prolonged AI therapy for endometriosis, especially in those with pre-existing metabolic risk factors such as obesity, insulin resistance, or a family history of diabetes [198,199].

#### 5.2.5. Hormonal Therapy and the Microbiota: An Underexplored Interaction

Hormonal therapies for endometriosis are not microbiota neutral. Because first- and second-line hormonal agents converge on either a hypoestrogenic state (GnRH agonists and antagonists, aromatase inhibitors, prolonged progestin exposure) or an altered estrogen profile (combined estrogen–progestin contraceptives), and because host estrogenic tone reciprocally shapes the microbiota (Section 3), effective hormonal suppression is mechanistically expected to produce parallel shifts in the gut and reproductive-tract microbiomes [80,84]. This interaction remains under-explored in endometriosis specifically, but the following considerations are supported by the broader estrogen–microbiome literature.

Combined estrogen–progestin contraceptives have been associated with shifts in gut microbiota diversity and composition, with the direction of effect varying by formulation and host factors [80,82]; effects on the vaginal microbiota are more consistent, with stabilization of *Lactobacillus* dominance reported in users [19,130]. Whether these shifts attenuate or potentiate endometriosis-associated dysbiosis is unknown, and the question is clinically relevant given the frequency and duration of contraceptive use.

Sustained hypoestrogenism induced by GnRH agonists or antagonists would be expected to recapitulate features of the post-menopausal microbiota, including reduced vaginal *Lactobacillus* dominance and altered gut β-glucuronidase activity [78,80]. Aromatase inhibitors produce an even more profound estrogen depletion and would be predicted to amplify such estrogen-dependent microbiota shifts. To date, no dedicated longitudinal study has profiled the gut, vaginal, or peritoneal microbiota in women receiving these therapies for endometriosis.

Taken together, hormonal therapy–microbiota interactions represent a coherent but largely unstudied dimension of endometriosis management. They are directly relevant to the model proposed in Section 4.3: if microbiota composition co-determines circulating bioactive estrogen through the estrobolome, then a hormonal therapy that also shifts the microbiota will exert an integrated rather than a purely pharmacological effect on host estrogenic tone. Dedicated trials combining serial microbiota sampling with pharmacokinetic and clinical outcomes are needed to test whether microbiota-targeted co-interventions could potentiate hormonal therapy, mitigate its adverse effects, or reduce post-treatment recurrence.

### 5.3. Emerging Therapies for Endometriosis

Emerging therapeutic strategies for endometriosis increasingly focus on targeting upstream mechanisms involved in disease pathophysiology, including immune dysregulation, inflammation, and microbiota alterations. These approaches aim to complement or potentially overcome the limitations of conventional hormonal therapies.

Immunomodulatory approaches for endometriosis aim to correct the complex immune dysfunctions characteristic of the disease, including impaired NK cell cytotoxicity, M2 macrophage polarization, and elevated pro-inflammatory cytokines such as TNF-α, IL-1β, and IL-6. These strategies also address dysregulation of neutrophils, dendritic cells, T/B lymphocytes, complement activation, and immune checkpoint pathways. Biologic agents (e.g., TNF-α antagonists—infliximab, etanercept, adalimumab, IL-1 receptor antagonists—anakinra, JAK/STAT inhibitors—tofacitinib) have shown promise in endometriosis by reducing lesion size, inflammatory cytokine levels, and oxidative stress. However, clinical application remains limited, and future directions include immune cell therapies, complement modulation, and nanotechnological approaches. Large-scale clinical studies are needed to validate the effectiveness and safety of these novel immunotherapies [133,200,201,202].

Neovascularization is essential for the establishment and proliferation of endometriotic lesions, as the survival and growth of ectopic endometrial tissue depend on the development of new blood vessels driven primarily by vascular endothelial growth factor (VEGF) signaling [58,203]. Antiangiogenic therapies targeting VEGF and its receptor (VEGFR) have demonstrated significant efficacy in animal models, consistently reducing lesion size and weight without compromising ovarian follicle number or function [204,205]. Agents such as bevacizumab, pazopanib, and sunitinib suppress VEGF-mediated angiogenesis, leading to decreased lesion vascularization and proliferation [206]. Dopamine agonists, including bromocriptine, cabergoline, and quinagolide, are promising candidates for antiangiogenic therapy in endometriosis. These agents downregulate proangiogenic pathways by inhibiting VEGF secretion and VEGFR-2 activation, reducing nerve fiber density within lesions, and decreasing the invasive and angiogenic properties of endometrial stromal cells. Importantly, dopamine agonists maintain ovulation and oocyte quality, a critical advantage for women seeking fertility preservation, as demonstrated in both animal and early human studies. However, the integration of antiangiogenic therapies into routine clinical practice awaits confirmation of long-term safety and efficacy through well-designed prospective human trials [207,208,209,210].

### 5.4. Probiotics, Postbiotics, and Dietary Interventions as Metabolic Adjuvants in Endometriosis

Microbiota-targeted interventions represent a particularly promising therapeutic avenue, given their ability to simultaneously modulate inflammation, immune responses, and metabolic pathways. Probiotic supplementation represents a mechanistically sophisticated therapeutic approach for endometriosis, with Lactobacillus-based formulations demonstrating multifaceted efficacy in reducing endometriosis-associated pain, modulating immune responses, and improving metabolic parameters in both preclinical models and clinical trials [211,212]. The therapeutic mechanisms of probiotics extend beyond simple microbial colonization to include the production of anti-inflammatory β-carboline compounds by species such as *Lactobacillus crispatus*, which suppress nuclear factor κB (NF-κB) and interferon signaling pathways, thereby dampening the chronic inflammatory milieu characteristic of endometriosis [213]. Furthermore, peptidoglycan derived from *Lactobacillus rhamnosus* and *Lactobacillus acidophilus* has been shown to suppress TLR2/1-mediated inflammation in endometrial epithelial cells, acting as a TLR2/1 antagonist and reducing the expression of pro-inflammatory cytokines, including TNF-α, IL-6, and IL-1β [214]. Probiotics also modulate pain perception through the gut-brain axis by targeting pain receptors, including TRPV1, cannabinoid (CB1, CB2), opioid (mu, kappa), and serotonin (5-HT) receptors, thereby alleviating visceral and neuropathic pain hypersensitivity [215]. The metabolic benefits of probiotic supplementation are particularly relevant for endometriosis patients, as specific strains including *Lactobacillus plantarum* S9, *Lactobacillus rhamnosus* Lb102, and *Bifidobacterium animalis* ssp. lactis Bf141 significantly improves glycemic control by reducing fasting plasma glucose, insulin levels, and HOMA-IR, while also favorably modulating lipid profiles through decreases in total cholesterol, LDL-cholesterol, and triglycerides [216,217,218]. These metabolic improvements are achieved through multiple mechanisms, including reduction of systemic inflammation via inhibition of the NF-κB pathway, enhancement of insulin sensitivity, and modulation of gut microbiota composition to increase beneficial bacteria such as Blautia, Roseburia, and Ruminococcus [216,219].

Postbiotics, defined as preparations of inanimate microorganisms and/or their components that confer a health benefit on the host, represent the next generation of microbiome-based therapeutics and offer unique advantages, including enhanced stability, safety, and defined mechanisms of action compared to live probiotics [220,221]. These preparations, comprising heat-inactivated microorganisms, bacterial metabolites such as short-chain fatty acids (SCFAs), peptidoglycans, exopolysaccharides, and bacterial cell wall components, exert metabolic and anti-inflammatory benefits through multiple interconnected mechanisms [222,223,224]. In the context of endometriosis, postbiotics may address both the inflammatory environment and metabolic dysregulation characteristic of the disease through several key pathways [112]. Postbiotics enhance intestinal barrier integrity by upregulating tight junction proteins, including ZO-1, occludin, and claudin-1, thereby reducing intestinal permeability and preventing the translocation of bacterial lipopolysaccharide LPS that drives systemic inflammation [225,226,227,228]. They modulate immune responses by suppressing the TLR4/NF-κB and MAPK signaling pathways, reducing pro-inflammatory cytokine secretion (TNF-α, IL-6, IL-1β), and promoting regulatory T-cell responses while balancing Th1/Th2 and Treg/Th17 immune profiles [229]. Postbiotics also activate the Nrf2/ARE antioxidant pathway, upregulating key antioxidant enzymes (NQO1, HO-1) and reducing oxidative stress, which is particularly relevant given the oxidative damage observed in endometriotic lesions. Furthermore, postbiotics directly influence gut microbiota composition, increasing beneficial bacteria and enhancing SCFA production, which creates a favorable metabolic environment [230].

SCFAs, particularly butyrate, propionate, and acetate, represent critical mediators linking gut microbiota to endometriosis pathophysiology and metabolic regulation. Patients with endometriosis demonstrate reduced abundances of butyrate-producing microbiota compared to healthy controls, and this deficiency contributes to disease progression. Butyrate exerts anti-endometriosis effects through multiple mechanisms: it enhances ferroptosis sensitivity in endometriotic stromal cells via the FFAR2/PPAR-γ/PINK1/Parkin-mediated mitophagy pathway, thereby promoting cell death in ectopic lesions; it acts as a histone deacetylase (HDAC) inhibitor, modulating gene expression and suppressing lesion proliferation; and it reduces immune dysregulation and chronic inflammation [231,232,233].

Acetate similarly ameliorates endometriosis by activating the JAK1/STAT3 signaling pathway, driving macrophage polarization toward the pro-inflammatory M1 phenotype (characterized by increased iNOS/CD86 expression and decreased Arg1/CD206 expression), which contrasts with the M2 macrophage dominance typically observed in endometriotic lesions. Acetate supplementation through fecal microbiota transplantation from healthy donors significantly reduces ectopic lesion volume and weight while enhancing intestinal barrier integrity by upregulating tight junction proteins and reducing intestinal permeability [234]. The anti-inflammatory and anti-proliferative effects of SCFAs are crucial for counteracting the cell proliferation, immune surveillance escape, and invasive metastasis that characterize endometriosis development [211,233].

Dietary interventions complement these microbiome-targeted approaches and represent evidence-based, non-invasive adjuvant strategies for managing endometriosis-related symptoms and metabolic dysfunction [235,236]. The Mediterranean diet, characterized by high consumption of fruits, vegetables, whole grains, legumes, nuts, olive oil, and fish, has demonstrated particular promise in reducing endometriosis symptoms and improving metabolic outcomes through multiple synergistic mechanisms. Also, anti-inflammatory dietary patterns work by providing substrates for beneficial microbial metabolism, including dietary fiber that promotes SCFA production by gut bacteria, thereby creating a favorable intestinal environment. These diets reduce systemic inflammation through high intake of antioxidant-rich foods containing polyphenols, vitamins C, D, and E, which neutralize reactive oxygen species and reduce oxidative stress in endometriotic lesions [237,238,239].

Dietary modulation of estrogen metabolism represents another critical mechanism, as increased intake of polyunsaturated fatty acids (PUFAs), particularly omega-3 fatty acids from fish and plant sources, influences the estrobolome, the collection of gut bacteria capable of metabolizing estrogens, thereby reducing circulating estrogen levels that drive endometriosis progression [130,238]. Omega-3 PUFAs, specifically eicosapentaenoic acid (EPA) and docosahexaenoic acid (DHA), exert direct anti-endometriosis effects by suppressing cystic lesion formation through the 12/15-lipoxygenase pathway, which produces anti-inflammatory mediators including 12/15-HEPE and EPA-derived resolvin E3 [240,241].

The low-FODMAP diet has shown particular efficacy in reducing gastrointestinal symptoms commonly experienced by endometriosis patients, including bloating, abdominal pain, and altered bowel habits, by reducing fermentable substrates that can exacerbate intestinal inflammation and permeability. A recent randomized controlled crossover trial demonstrated that 60% of women with endometriosis responded to a 28-day low-FODMAP diet compared to only 26% on a control diet, with significant improvements in overall gastrointestinal symptom scores, abdominal pain, bloating, stool form, and quality of life [242,243]. The mechanism underlying these benefits relates to the reduction of fermentable carbohydrates that can trigger visceral hypersensitivity, a shared pathophysiological feature in both endometriosis and functional gastrointestinal disorders [244].

Dietary interventions also improve insulin sensitivity and glucose metabolism through multiple pathways: reduction of refined carbohydrates and processed foods decreases insulin resistance; increased fiber intake slows glucose absorption and improves glycemic control; and consumption of foods with low glycemic index prevents postprandial hyperglycemia and hyperinsulinemia [235,238].

Overall, current therapeutic strategies for endometriosis remain largely focused on symptom control rather than disease modification. The growing recognition of the microbiota as a regulator of immune, endocrine, and metabolic pathways offers a novel perspective on disease management. Integrating microbiota-targeted approaches with conventional therapies may provide a more comprehensive and mechanism-based strategy, although robust clinical evidence is still required to support their routine use.

## 6. Conclusions

Endometriosis is a multifactorial, chronic inflammatory disease driven by a complex and self-sustaining interaction between endocrine imbalance, immune dysfunction, and persistent inflammatory signaling. While traditional models have focused primarily on retrograde menstruation and hormonal dependence, emerging evidence supports a more integrated perspective in which the microbiota acts as a critical modulator of disease susceptibility and progression.

The microbiota-endometriosis axis provides a unifying framework linking gut dysbiosis, immune activation, and altered estrogen metabolism. Through mechanisms such as increased intestinal permeability, lipopolysaccharide-driven inflammation, immune cell reprogramming, and estrobolome-mediated estrogen reactivation, microbial imbalance may contribute to the establishment of a pro-inflammatory and hyperestrogenic microenvironment that favors lesion persistence and growth. Importantly, this relationship appears to be bidirectional, with endometriotic lesions further influencing microbial composition, thereby reinforcing a pathological feedback loop. Despite these advances, the current body of evidence remains limited by heterogeneity in study design, small sample sizes, and a predominance of preclinical data. As such, the microbiota should be regarded not as a primary etiological factor, but as a dynamic and potentially modifiable contributor to disease pathophysiology.

From a therapeutic perspective, microbiota-targeted interventions, including probiotics, postbiotics, short-chain fatty acids, and dietary modulation, represent promising adjunctive strategies that extend beyond conventional hormonal suppression. These approaches have the potential to simultaneously modulate inflammation, restore immune balance, and influence estrogen metabolism, addressing key mechanisms underlying disease persistence. However, their clinical applicability requires validation through robust, large-scale randomized controlled trials.

In conclusion, integrating the microbiota into the conceptual model of endometriosis advances our understanding of the disease from a purely gynecological condition to a systemic disorder involving immune-endocrine-metabolic interactions. This paradigm shift opens new avenues for personalized and mechanism-based therapies, with the potential to improve long-term outcomes and quality of life for affected patients.

## Figures and Tables

**Table 1:** Key pathogenic pathways involved in the establishment and persistence of endometriosis.

Pathway	Principal Mechanisms	Key Molecular Players	Clinical Implications	References
Local estrogen excess	- intracrine estradiol biosynthesis within ectopic lesions; - impaired estradiol inactivation; - relative independence from systemic ovarian estrogen	↑ aromatase (CYP19A1) ↑ StAR ↓ 17β-HSD2 ↑ estradiol	- promotes lesion growth, proliferation, and persistence; - supports therapies targeting estrogen synthesis (e.g., aromatase inhibitors) and signaling	[5,22,23,24,25,26,27,28]
Altered ER signaling	- ERβ overexpression with ERα suppression; - epigenetically driven receptor imbalance; - anti-apoptotic and pro-inflammatory signaling; - amplification of prostaglandin-mediated estrogen synthesis	↑ ERβ ↓ ERα ↑ COX-2; ↑ PGE2 ↑ aromatase	- enhances inflammatory signaling and apoptosis resistance; - suggests potential for receptor-specific approaches, particularly targeting ERβ	[29,30,31,32,33]
Progesterone resistance	- reduced PR-B expression; - PR-A predominance; - epigenetic silencing of progesterone signaling; - impaired decidualization and estrogen detoxification; - failure to suppress NF-κB	↓ PR-B ↑ PR-A/PR-B ratio ↓ 17β-HSD2 ↓ HOXA10 ↑ NF-κB	- reduces responsiveness to progestin-based therapies; - contributes to chronicity and recurrence, highlighting the need for alternative or combination treatments	[5,21,34,35,36,37]
Immune Evasion	- reduced NK cell cytotoxicity; estrogen-mediated immune suppression; - macrophage functional reprogramming; immune checkpoint activation	↓ NK cell activity ↑ PD-1/PD-L1 ↑M2-like macrophages ↓ cytotoxic T/NK signaling	- enables survival of ectopic endometrial cells and lesion persistence; - supports immunomodulatory strategies to restore immune surveillance	[5,38,39,40,41,42,43,44,45]
Chronic Inflammation	- sustained cytokine and chemokine production; - NF-κB and MAPK pathway activation; - estrogen–PGE2 feed-forward inflammatory loop; - nociceptor sensitization	↑ IL-1β IL-6 TNF-α ↑ CCL2/CCL5 ↑ COX-2 ↑ PGE2	- drives pain, lesion progression, and symptom severity; - provides rationale for anti-inflammatory and immune-targeted therapies	[5,21,23,24,46,47,48,49,50]
Angiogenesis	- hypoxia-driven neovascularization; - HIF-1α activation; - cytokine-amplified VEGF expression; - macrophage-mediated vascular remodeling	↑ HIF-1α ↑ VEGF ↑ pro-angiogenic macrophages	- facilitates lesion vascularization and expansion; - supports exploration of antiangiogenic therapies as disease-modifying approaches	[51,52,53,54,55,56,57,58]
Fibrosis	- persistent inflammation and tissue injury; - fibroblast and myofibroblast activation via EMT, FMT, and MMT; - excessive ECM deposition; - neuro-immune-fibrotic niche formation	↑ TGF-β ↑ activin A ↑ CTGF ↑ S1P ↑ myofibroblasts ↑ ECM	- contributes to chronic pelvic pain, organ dysfunction, and infertility; - associated with advanced disease and surgical complexity	[49,59,60,61,62,63,64,65,66]
Etiopathogenesis	- retrograde menstruation with permissive genetic, epigenetic, and immune context; - stem/progenitor cell contribution; coelomic metaplasia; - lesion persistence and recurrence	↑ eMSCs ↑ epigenetic regulators altered immune surveillance	- explains lesion persistence and recurrence after treatment; - supports therapies targeting stem cell populations and underlying disease mechanisms	[5,20,21,38,67,68,69,70]

Abbreviations: 17β-HSD2—17β-hydroxysteroid dehydrogenase type 2; CCL2—CC motif chemokine ligand 2 (monocyte chemoattractant protein-1); CCL5—CC motif chemokine ligand 5 (RANTES); COX-2—cyclooxygenase-2; CTGF—connective tissue growth factor; ECM—extracellular matrix; EMT—epithelial-to-mesenchymal transition; ERα—estrogen receptor alpha; ERβ—estrogen receptor beta; eMSCs—endometrial mesenchymal stem/stromal cells; FMT—fibroblast-to-myofibroblast transdifferentiation; HIF-1α—hypoxia-inducible factor 1 alpha; HOXA10—homeobox A10; IL-1β—Interleukin-1 beta; IL-6—Interleukin-6; MAPK—mitogen-activated protein kinase; MMT—macrophage-to-myofibroblast transition; NF-κB—nuclear factor kappa B; NK cells—natural killer cells; PD-1—programmed cell death protein 1; PD-L1—programmed death-ligand 1; PGE2—prostaglandin E2; PR-A—progesterone receptor A; PR-B—progesterone receptor B; S1P—sphingosine-1-phosphate; StAR—steroidogenic acute regulatory protein; TGF-β—transforming growth factor beta; TNF-α—tumor necrosis factor alpha; VEGF—vascular endothelial growth factor.

**Table 2:** Summary of current and emerging therapeutic strategies in endometriosis and their mechanisms, clinical effects, limitations, and key references.

Therapeutic Class	Representative Agents/Interventions	Mechanism of Action	Key Clinical Effects	Limitations and Adverse Effects
NSAIDs	Ibuprofen, Naproxen	Inhibition of cyclooxygenase (COX) enzymes and prostaglandin synthesis	Reduction of pain and inflammation	Limited evidence No effect on disease progression
Combined oral contraceptives	Ethinyl estradiol + progestins	Suppression of ovulation and estrogen production; anti-proliferative effects	Reduction of dysmenorrhea and pelvic pain	Recurrence after discontinuation Metabolic effects
Progestins	Dienogest, norethindrone acetate, LNG-IUS	Decidualization and atrophy; anti-inflammatory effects	Effective long-term pain control	Irregular bleeding Variable metabolic impact
GnRH agonists/ antagonists	Leuprolide, goserelin, elagolix, relugolix	Suppression of the hypothalamic–pituitary–gonadal axis	Reduction in pain and lesion activity	Hypoestrogenic effects Limited duration High cost
Aromatase inhibitors	Letrozole, anastrozole	Inhibition of local estrogen biosynthesis	Effective in refractory cases	Bone loss Metabolic disturbances
Immunomodulators (emerging)	TNF-α inhibitors, IL-1 antagonists, JAK inhibitors	Modulation of immune response; reduction of cytokines	Potential reduction in inflammation and lesion size	Limited clinical evidence Safety concerns
Antiangiogenic therapies (emerging)	Bevacizumab, dopamine agonists	Inhibition of VEGF signaling and angiogenesis	Reduction in lesion vascularization	Limited human data Safety is not fully established
Probiotics	*Lactobacillus* spp., *Bifidobacterium* spp.	Modulation of microbiota; immune regulation; anti-inflammatory effects	Improvement of pain and inflammation	Heterogeneity of strains Limited trials
Postbiotics	SCFAs and microbial metabolites	Enhancement of barrier integrity; modulation of NF-κB and TLR pathways	Reduced inflammation and oxidative stress	Emerging evidence Limited clinical validation
Short-chain fatty acids	Butyrate, acetate, propionate	Epigenetic regulation (HDAC inhibition); immune modulation	Reduction in lesion progression (preclinical)	Mainly experimental data
Dietary interventions	Mediterranean diet, low-FODMAP, omega-3	Modulation of microbiota and estrobolome; anti-inflammatory effects	Improvement of symptoms and metabolic profile	Variable adherence Interindividual variability

**Table 3:** Pharmacologic Agents for the Treatment of Endometriosis.

Class of Drugs	Representative Agents	Mechanism of Action	References
NSAIDs	Ibuprofen Naproxen	Inhibit COX-1/COX-2 enzymes, reducing prostaglandin synthesis, thereby decreasing inflammation and pain signaling.	[49,144,145]
Combined Oral Contraceptives	Ethinyl estradiol + Norethindrone Ethinyl estradiol + Levonorgestrel	Suppress ovulation via negative feedback on the hypothalamic-pituitary axis; induce decidualization and atrophy of endometrial and ectopic tissue; reduces estrogen-driven proliferation.	[49,164,165,166]
Progestins	Norethindrone acetate, Medroxyprogesterone acetate Dienogest Levonorgestrel IUS	Induce decidualization and atrophy of endometrial tissue; suppress estrogen-induced mitosis; inhibit angiogenesis, neurogenesis, and inflammatory pathways; reduce local estrogen receptor expression.	[21,145,167]
GnRH Agonists	Leuprolide Goserelin Nafarelin	Downregulate pituitary GnRH receptors, suppress gonadotropin release, and induce a hypoestrogenic state, leading to regression of endometriotic lesions.	[149,166,168,169]
GnRH Antagonists	Elagolix Relugolix Linzagolix	Competitively inhibit pituitary GnRH receptors, rapidly suppress gonadotropin secretion, and induce a hypoestrogenic state, leading to regression of lesions.	[5,164,165,170]
Androgenic Steroid	Danazol	Inhibits pituitary gonadotropin secretion and ovarian steroidogenesis; direct antiproliferative effect on endometriotic tissue; induces a high-androgen, low-estrogen environment.	[144,145,164]
Aromatase Inhibitors	Letrozole Anastrozole	Inhibit aromatase enzyme, blocking conversion of androgens to estrogens; reduce local and systemic estrogen production, suppressing growth of endometriotic lesions.	[145,166,170]

## Data Availability

No new data were created or analyzed in this study. Data sharing is not applicable to this article.
